# The impact of entrepreneurial education on the propensity of business students to support new ventures: A moderated mediation model

**DOI:** 10.3389/fpsyg.2022.1046293

**Published:** 2022-11-22

**Authors:** Shahzad Khuram, Hina Ahmed, Shahzad Ali

**Affiliations:** Superior College Lahore, Lahore, Pakistan

**Keywords:** entrepreneurial education, financial self-efficacy, intention toward venture capital, government support, business school

## Abstract

This research aims to investigate the role of financial self-efficacy in mediating the relationship between entrepreneurial education and venture capital intention, as well as the moderating influence of government support on the relationship between financial self-efficacy and venture capital intention. The target population consists of Lahore students from public and private universities who have already studied entrepreneurship. Based on the 250 responses to the online survey the findings show the mediating role of financial self-efficacy between entrepreneurial education and intention toward venture capital. Moreover, it also indicates the moderated effect of government support on the relationship between financial self-efficacy and intention toward venture capital. This study will not only help the curriculum committees in business schools to design entrepreneurial education outlines that enable the students to explore the different financing modes by including financial knowledge to cope with financial challenges but also to the government officials in devising financing plans accompanied by their expertise in the development of a business.

## Introduction

Entrepreneurship has gained popularity as a result of its positive impact on global economic and social growth. It is regarded as a creative and innovative process capable of raising yield, generating new opportunities, reinvigorating and expanding businesses, promoting welfare programs, and strengthening the economy of a nation (Guerrero et al., 2008). Entrepreneurs contribute a crucial role in the progress and prosperity of a country’s economy and the well-being of its citizens (Iakovleva et al., 2014), enhance employability, and bring innovation (Kelley et al., 2011). Kelley (2017) claimed that there are more than 582 million entrepreneurs worldwide including 274 million female entrepreneurs. As more and more individuals are involved in entrepreneurial activities around the world the sizes of businesses are shrinking (Gallagher, 2022). No doubt, a growing trend has been observed in entrepreneurial activities but developing countries like Pakistan are still facing the challenges of lower investment rates in new ventures. Although, many people maintain cultivating a fantastic business concept, but lack the confidence to implement it and give it birth due to the dangers associated with start-ups (Junejo et al., 2022). This lack of confidence is mostly because of their inability to achieve their financial goals (Klimas et al., 2021). Many researchers have focused on venture capital firms, which are among external startup investors, because of their essential role in the formation of new ventures (Kaplan and Strömberg, 2003). In addition to financial contributions, venture capital firms give significant intangible assets in form of knowledge and connections (Wang and Zhou, 2004). This aspect of venture capital investment is critical as new enterprises usually lack adequate financial and intangible capabilities, such as existing knowledge and experience, that need to thrive. However, only a tiny percentage of businesses have been successful in obtaining venture capital investment, and the time of obtaining investments ranges from early to late in a startup’s development (Gompers and Lerner, 2001). While the majority of venture-funded companies fail, others, like the six largest United States companies by market capitalization [Apple, Microsoft, Alphabet (Google), Amazon, Facebook, and Tesla] obtain the majority of their early external funding from venture capitalists. These businesses were still unknown and speculative when they received their first venture capital financing (Gornall and Strebulaev, 2021). Venture capital firms were the first to fund global success stories including Intel, Oracle, Skype, Federal Express, Cisco, AMD, and 3Com (Kenney et al., 2002). Venture finance has piqued the curiosity of governments all around the world. This fascination arises in part from the pivotal role that venture capitalists have played in the expansion of some of the world’s most powerful businesses (Brander et al., 2015).

With almost 229 million people, Pakistan is the fifth-most populous country in the world.^1^ It is facing an issue of unemployment, which is about 4.4%. Pfeiffer and Reize (2000) suggested one possible solution to this problem is increasing the number of new startups. Credit is one of the most crucial aspects to think about when beginning a new business or enterprise, and easy credit is important not only in Pakistan but around the world (Carney and Gedajlovic, 2002; Ahmad and Hoffmann, 2008). Furthermore, according to the global entrepreneurship monitor (GEM, 2019), a group of regional country teams that conducts survey-based entrepreneurial research and is primarily connected to leading universities of higher education, although the choice of entrepreneurship as a good career opportunity is high (80.15%) in Pakistan, yet due to the higher probability of fear of failure (54.16%) the intention toward the entrepreneurship is very low (27.90%). Shinnar et al. (2018) claimed that university graduates who have obtained entrepreneurship education are more likely to pursue entrepreneurial careers. Realizing the need of the hour, few universities in Pakistan are entirely dedicated to the study of entrepreneurship (Li et al., 2021).

Liñán and Fernandez-Serrano (2014) described “that entrepreneurial education can be found in the whole set of education and training activities that intend to perform entrepreneurial behaviors, or some of the elements that affect that intention, such as entrepreneurial knowledge, the desirability of the entrepreneurial activity, or its feasibility.” Through entrepreneurial knowledge, the students are taught the ways to prepare financial feasibility that enhances their financial handling capacity. Financial self-efficacy (FSE), or one’s belief in one’s competence to handle money, influences money-handling behavior. Lusardi et al. (2010) described financial education, as an education that enables people to make smarter financial decisions, understand their obligations and rights as the users of financial products, and better risk management. Brandon and Smith (2009) described FSE, as one’s belief in one’s competence to handle money with financial awareness. According to their results, the more their financial self-efficacy of knowledge, the more efficiently they can use money, resulting in improved behavior.

The government’s initiatives to boost entrepreneurial activities such as practical courses (Rehman and Roomi, 2012) and monetary support to launch their start-up under a 10-year prospective development plan support new entrepreneurs to make them more beneficial to society at large. In addition, several initiatives have been formed, such as the National productivity organization, Pakistan poverty alleviation fund, Karandaaz, and Rural support programs. Furthermore, Pakistan’s public and private sectors are working together to encourage entrepreneurship (Nasir et al., 2019). Such initiatives by the government, support new ventures to look for the financing choices such as venture capital.

The study’s major goal is to examine the influence of entrepreneurial education on financial self-efficacy as well as venture capital intention and to examine the mediating role of financial self-efficacy between entrepreneurial education and venture capital intention. Moreover, the study is also aimed to explore the moderation effect of government support between financial self-efficacy and intention toward venture capital. The study therefore will help the curriculum committees in business schools to design such entrepreneurial education outlines that enables the students to explore the different financing modes to establish or expand their businesses and to include such financial knowledge that may enable them to cope with financial challenges. Additionally, this study will assist government officials in creating financing strategies that might not only finance but also offer their knowledge in business growth.

The next section of the study will review, the literature related to entrepreneurial education, financial self-efficacy, intention toward venture capital, and government support that will be resulting in a model for testing. This will be followed by the methodology and data analysis. In the end, the theoretical and practical implications, limitations, and future directions will be written.

## Literature review

### Theory of planned behavior

The theory of planned behavior (TPB), which is a continuation of the theory of reasoned action (Ajzen and Fishbein, 1975), serves as the theoretical foundation for this study. According to TPB, people use the knowledge at their disposal to make informed decisions about engaging in particular behaviors. There have been several models proposed to study these intentions. However, among all the theories, Ajzen’s social psychology theory of planned behavior (TPB), is the one that is most frequently employed to evaluate intents (Zaremohzzabieh et al., 2019). According to Entrialgo and Iglesias (2016), the TPB has frequently been employed to explain the intentions of university students. Scholars from a variety of disciplines have thoroughly studied the TPB, and its fame increases the predictive and explanatory potential in education (Maheshwari and Kha, 2022). It has established a sufficient theoretical foundation for past studies on entrepreneurship education and, to some extent, can be claimed to influence intention formation (Su et al., 2021). Intention deviates over time (Su et al., 2021) and universities may influence such changes in their students through education (Maheshwari and Kha, 2022), potentially increasing students’ financial self-efficacy (Bell and Bell, 2016). Moreover, the relationship between self-efficacy and intentions has been linked to the development of entrepreneurs (Li et al., 2021). This study also used the TPB perspective in conjunction with government support to extend the TPB framework and explain the effect of such support on university students’ intentions toward venture capital (Su et al., 2021).

### Relationship between entrepreneurial education and intention toward venture capital

Entrepreneurial education is a source of equipping students with the desired abilities to deal with the issues that have to be faced during a normal course of business (González-López et al., 2019). Entrepreneurial education consists of “any pedagogical [program] or process of education for entrepreneurial attitudes and skills” (Fayolle et al., 2006). After passing through a long history, it has become a phenomenon now (Katz, 2003; Kuratko, 2005). It has a different type that focuses on the different phases of progress (Mcmullan and Long, 1987; Gorman et al., 1997; Bridge et al., 1998). Moreover, different modes of entrepreneurial education have been identified by academics, each of which is tailored to a unique audience (Jamieson, 1984; Liñán, 2004). Education to manage finance and the modern source of finance will enable the entrepreneur to make smart financial decisions and hence achieve business goals (Bruhn and Zia, 2013). Fatoki (2014) concludes that education on entrepreneurial finance is an important knowledge and key to the success of entrepreneurs. Bruhn and Zia (2013) and Dahmen and Rodríguez (2014), have further highlighted the lack of knowledge of entrepreneurial finance among young entrepreneurs which usually causes financial issues in their new ventures. In recent years, the difficulties in getting finance for early-stage ventures have emerged new sources of funding that include; crowdfunding, accelerators, private and government venture capital, peer-to-peer business lending, etc. (Bruton et al., 2015; Block et al., 2018). Sahlman (1990) described that the professional investment management activity of venture capital aims to raise money from wealthy people and institutional investors to invest in new enterprises with risky concepts but great growth potential. The average duration of the raised fund is 7–10 years. Venture capitalists choose portfolio companies, mentor, supervise, and give value-added services during this time (Sapienza, 1992; Lerner, 1995), After being compensated by the investors, they eventually quit the companies, sharing the profits with the institutional investors. Venture capitalists are widely assumed as the perceived interceders who provide finance to the early-stage and hi-tech businesses that may otherwise struggle to get funding from traditional sources (Gompers and Lerner, 2001). In light of the above-cited literature the study hypothesizes the following;

*H1*: Entrepreneurial education has a significant impact on intention toward venture capital.

### The mediating role of FSE

FSE describes the person’s confidence in his ability to get, consume, and make smart financial choices, to handle a situation where financial management became a challenge (Amatucci and Crawley, 2011; Ghosh and Vinod, 2017). Financial self-efficacy is linked and associated with social cognitive theory, which claims that all aspects of an individual’s life are influenced by self-efficacy includes; motives, choices, devotion in compliance to a task, positive and negative thoughts, and the level of persistence with which they dealt with the problems. The higher the recognizability of one’s self-efficacy, the higher it will have an impact on one’s achievements, feeling, behavior, and self-motivation (Bandura, 1991, 2005). This variable was discovered to mediate the relationship between many variables and the execution of intentional behaviors in certain domains across time. Self-efficacy is not at all an invariable term (Ng and Lucianetti, 2016), it is shaped by several factors that may be internal or external such as goals achievements (Du et al., 2020), creativity education (Mathisen and Bronnick, 2009), and societal assistance (Mathisen, 2011). Students who acquire the necessary financial knowledge and expertise are also self-assured in their abilities to close deals successfully. Self-efficacy can be nourished with educational processes as it is developed on the information of the four sources that are; real performance, emotional stimulation, mediated learning, and social encouragement (Wood and Bandura, 1989). The size of the association between self-efficacy and educational excellence is three times that of the relationship between present and former educational excellence (Gore, 2006). In this study, the argument has been developed that entrepreneurial education enhances financial literacy and boosts the student’s financial self-efficacy. As a result, financial self-efficacy is linked to entrepreneurial education and these arguments develop the following hypothesis.

*H2a*: Entrepreneurial education has a significant impact on FSE.

Campopiano et al. (2017) described FSE as a person’s trust in his ability to obtain financial objectives. If the person’s level of trust is higher, he or she will be highly motivated to do anything necessary for the achievement of the desired goals, also, when it is related to somebody’s conduct in handling their finances. The higher the level of FSE the better it will be in managing financial challenges. In addition to the abilities that individuals possessed, In the process of cognitive thought to attain the intended action motivated by willpower, self-efficacy had an indirect part (Hejazi et al., 2009). McGee et al. (2009) stated that self-efficacy increased the likelihood of an individual’s intention being transformed into desired actions. Some other studies such as those (Farashah, 2013; Setiawan, 2014; Miranda et al., 2017) also verified the significance of self-efficacy in the development of intention among individuals. Based on the reasoning presented above this study hypothesizes the following:

*H2b*: Financial self-efficacy has a significant impact on intention toward the capital venture.

From the previous discussion in the literature, it is obvious that entrepreneurial education has an important role in the enhancement of financial self-efficacy and intention toward venture capital. Also, financial self-efficacy can influence an individual intention toward venture capital. So conceptually, financial self-efficacy can provide a mechanism for entrepreneurial education to influence intention toward venture capital. Some studies conducted in China and United States by (Zhao et al., 2005; Chen and He, 2011; Kassean et al., 2015) discovered that self-efficacy can mediate the relationship between entrepreneurial education and intention toward attitude. A related study conducted by Piperopoulos and Dimov (2015) involving students from British universities also revealed the mediating role of self-efficacy. Based on the arguments discussed in the literature so far, this study argues that there exists a mediating relationship of financial self-efficacy between entrepreneurial education and intention toward venture capital. Based on this argument the following hypothesis is developed.

*H2*: FSE significantly mediates the relationship between entrepreneurial education and intention toward venture capital.

### The moderating role of government support

Governments all over the world intervene in the private sector that is involved in financing early-stage ventures. Since the governments have always been active in controlling and financing state intervention in the private sector, and in controlling and financing transnational, it is not new. Verheul et al. (2002) proposed that governments should balance both the demand and supply aspects of entrepreneurship. The governments have acknowledged the importance of entrepreneurship and hence passed such laws to enhance the funding to early-stage businesses, for instance, United States Congress passed the small business innovation research (SBIR) in 1982, and the objective of this program was to boost the competitiveness among the Americans. The legislation made provisions for funding innovative small businesses (Lerner and Kegler, 2000; Cooper, 2003). By cultivating a national entrepreneurial culture, government programs lend legitimacy to entrepreneurship in society. This culture encourages people to start their firms by instilling a positive attitude toward entrepreneurship (Shinnar et al., 2012). Several European governments have set up venture capital funds for high-tech startups (Cumming and Johan, 2013; Cumming et al., 2017). Government programs can help entrepreneurs in developing countries achieve their goals. The national investment trust limited (NITL), a subsidiary of the Pakistani government, has formed Rs. 1 billion venture capital funds to encourage Pakistani startups. However, such initiatives taken by the government to encourage entrepreneurship will remain ineffective if individuals remain unable to recognize such funding opportunities. The potential entrepreneur should be able to recognize the existing entrepreneurial opportunity and must possess skills to make the maximum out of it (Shane and Venkataraman, 2000). Entrepreneurs’ self-perceived difficulty to launch their firms frustrates venture capitalists and economic development institutes (Griffiths et al., 2009). Therefore, besides government support, a certain level of financial self-efficacy, and intention toward the venture. Based on these arguments, this study postulates that government support can strengthen the relationship between financial self-efficacy and the intention toward venture capital. Based on this argument the following hypothesis is developed.

*H3*: Government support moderates significantly between financial self-efficacy and intention toward venture capital.

### Research model

See Figure 1.

**Figure 1:**
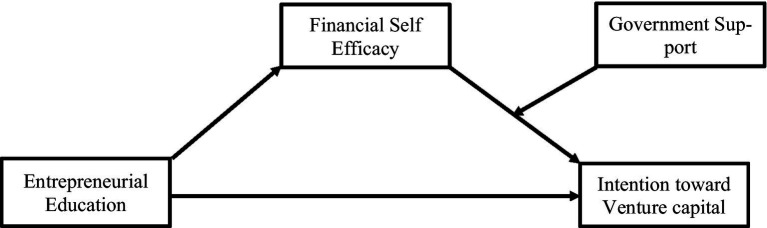
Research model.

## Materials and methods

### Questionnaire and measures

There were 29 questions in the questionnaire divided into five sections: (a) the demographic including, age, gender, qualification, and University/DAI., (b) entrepreneurial education, (c) financial self-efficacy, (d) the government support, and (e) intention toward venture capital. Based on the study of (Nguyen et al., 2011), entrepreneurial education was measured by a 4 items scale. A six-item scale adapted from research (Lown, 2011) on financial self-efficacy was used to assess it. The government support scale consisted of 11 questions taken from the study by (Korosec and Berman, 2006). For venture capital intention a 3 items scale from the study (Baber, 2020) was used. All the scales were adapted and measured on 5 Likert scales from strongly agree (5) to strongly disagree (1) except for financial self-efficacy which was measured on exactly true to not at all true scale (Table 1).

**Table 1:** Instruments.

Sr	Variable name	Items	Source
	Entrepreneurial education	4	Nguyen et al. (2011)
	Intention toward venture capital	3	Baber (2020)
	Financial self-efficacy	6	Lown (2011)
	Government support	11	Korosec and Berman (2006)

### Sample design and data collection

This study used an online survey questionnaire method and a deductive methodology. The study’s target audience was students of public and private universities who have already studied entrepreneurship. The selection of university students for this study was based on the fact that universities are regarded as the key cultural determinant (Li et al., 2021). The city of Lahore was chosen for data collection as it is the center of Pakistan’s educational system because it is home to some of the nation’s most prominent, renowned, and prestigious universities (Noor et al., 2020).

The list of universities offering business and management studies was obtained from the higher education commission website. These universities were separated into two clusters, i.e., the public and private sectors. For data gathering, a simple random sampling technique is employed in four universities from each cluster that were chosen based on the larger number of students enrolled in these universities. These selected universities also represent all other universities of Lahore as these were selected on enrolment criteria. The online questionnaire was shared with the targeted students through the HoDs and faculty members of the selected universities *via* WhatsApp groups. The students were communicated about the importance of this research through the introductory information in the online questionnaire. Moreover, they were also informed about the variables and their relationship to this study. 250 questionnaire responses in all were collected and analyzed using AMOS version 24.0. The questionnaire was completed between 28th March and 20th July 2022.

### Demographics

According to the results of the online survey, which included 250 participants, 77.2% of male and 22.8% of female participants responded to this survey. The low percentage of female respondents is because of their low rate of enrollment in business studies as compared to male students. 78.4% of the respondents were in between 21 and 25 years of age. 83.2% of the respondents were undergraduates.

## Data analysis

The hypothesized relationships were analyzed using structural equation modeling (SEM) generated through AMOS version 24.0. SEM also includes measurement error, and it can show the most accurate estimates of interacting influences, like mediation (Hair et al., 2014; Li et al., 2020; Cui et al., 2022).

### Model fit

**Figure 2:**
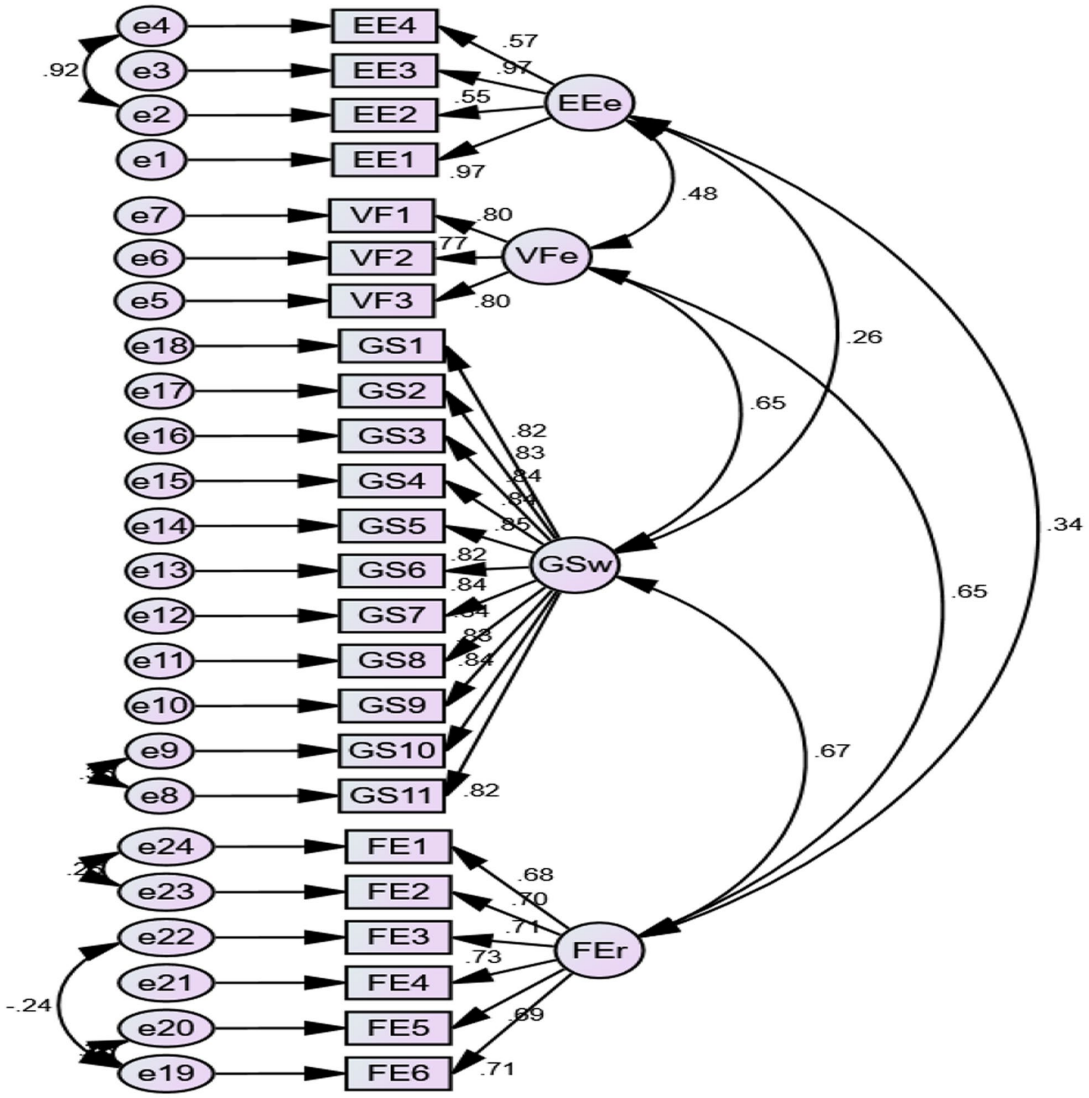
Model Fit. EE, entrepreneurial education; VF Intention toward venture capital; GS, government support; FE, financial self-efficacy.

Model fitness was accessed by performing confirmatory factor analysis (Nadeem et al., 2020), and the findings are reflected in Figure 2. Moreover, Table 2 below shows the criteria used to determine model fit. The measurement model fulfills the proposed threshold values (Hu and Bentler, 1999; Li et al., 2021), hence, demonstrating a good model fit.

**Table 2:** Model fit criteria.

Measurement	Estimate	Interpretation
CMIN/DF	1.849	Excellent
CFI	0.9459	Excellent
IFI	0.960	Excellent
NFI	0.916	Excellent
RMSEA	0.058	Excellent
PCLOSE	0.53	Excellent

### Descriptive statistics and correlations analyses

To study variables, means, standard deviations, and correlations were calculated. Results are shown in Table 3. According to the results derived from the data collected from the students of business schools who have already studied entrepreneurship, entrepreneurial education was significantly and positively connected with the intention toward venture capital (*r* = 0.514, *p* < 0.01) and financial self-efficacy (*r* = 0.346, *p* < 0.01). The financial self-efficacy was positively connected with government support (*r* = 0.601, *p* < 0.01) and intention toward venture capital (*r* = 0.546, *p* < 0.01). In addition, was positively connected with entrepreneurial education (*r* = 0.346, *p* < 0.01). As a result, the correlation analysis’ findings offered supportive evidence for the mediated-effects test that followed. In the current study, gender, age and qualification were also employed as control variables.

**Table 3:** Descriptive statistics and correlations analyses.

Variables	Mean	SD	1	2	3	4
EE	4.4180	0.68714				
GS	3.7833	0.95215	0.323^**^			
IVC	3.9813	0.86718	0.514^**^	0.584^**^		
FSE	3.8480	0.81732	0.346^**^	0.601^**^	0.546^**^	

*N* = 250. EE, entrepreneurial education; IVC, intention toward venture capital; GS, government support; FSE, financial self-efficacy.

** Correlation is significant at the 0.01 level (2-tailed).

### Convergent validity

Factor loading, Cronbach Alpha (CA), Composite reliability (CR), and Average variance extract (AVE) were used to assess Convergent Validity. The values in Table 4 represent convergent validity. The factor loading for the established structures should be greater than 0.60, according to Dash and Paul (2021). The majority of the items are greater than the minimum allowed value. Cronbach’s alpha values of all the variables are above 0.7 and hence represent good internal reliability of the scales (Cronbach, 1951). Additionally, Hair et al. (2021) showed that the values of CR and AVE were above the minimum acceptable thresholds of 0.70 and 0.50, respectively. The convergent validity was acceptable because all CA, CR, and AVE values fell within acceptable ranges.

**Table 4:** Convergent validity.

Variables	Items	Loading	CA	CR	AVE
Entrepreneurial education	EE1	0.972	0.891	0.863	0.630
	EE2	0.548			
	EE3	0.975			
	EE4	0.57			
Intention toward venture capital	IVC1	0.803	0.860	0.834	0.626
	IVC2	0.769			
	IVC3	0.803			
Government support	GS1	0.816	0.962	0.961	0.692
	GS2	0.839			
	GS3	0.827			
	GS4	0.841			
	GS5	0.839			
	GS6	0.822			
	GS7	0.849			
	GS8	0.837			
	GS9	0.842			
	GS10	0.826			
	GS11	0.816			
Financial self-efficacy	FSE1	0.708	0.860	0.856	0.500
	FSE2	0.695			
	FSE3	0.733			
	FSE4	0.715			
	FSE5	0.704			
	FSE6	0.682			

### Discriminant validity

Discriminant validity refers to the degree to which each latent variable in the study differs from other variables in the model (Hair et al., 2014). The discriminant validity was measured using the Heterotrait-Monotrait ratio (HTMT) and the Fornell and Larcker criterion. Table 5 compares the square roots of each AVE in the diagonal with the correlation coefficients (off-diagonal) for each construct in the associated rows and columns to demonstrate the establishment of discriminant validity as proposed by Fornell and Larcker (Hussain et al., 2021). Table 6 displays the HTMT ratios that are less than 0.85 (Henseler et al., 2016). The discriminant validity of every variable investigated is established as a result.

**Table 5:** Fornell and Larcker criteria.

	EE	IVC	GS	FSE
EE	0.794			
IVC	0.485	0.792		
GS	0.261	0.648	0.832	
FSE	0.342	0.652	0.671	0.706

**Table 6:** Heterotrait-Monotrait ratio (HTMT).

	EE	IVC	GS	FSE
EE	–			
IVC	0.594	–		
GS	0.345	0.653	–	
FSE	0.399	0.65	0.663	–

### Common method biased

There are various preventive, investigative, and corrective strategies available in the literature to be used to alleviate fears about the probability of common methods biases in the basic reported results. Harman’s single-factor test has been the most popular and largely used method to detect CMB (Aguirre-Urreta and Hu, 2019; Yong et al., 2021). This bias develops when any particular factor explains more than half of the overall variance (Podsakoff et al., 2012). To test CMB all the factors were combined to form a single factor and they collectively explain 31.733% of the variance. Which is within the acceptable threshold, i.e., <50%. Hence, there exists no CMB.

### Hypothesis testing

**Figure 3:**
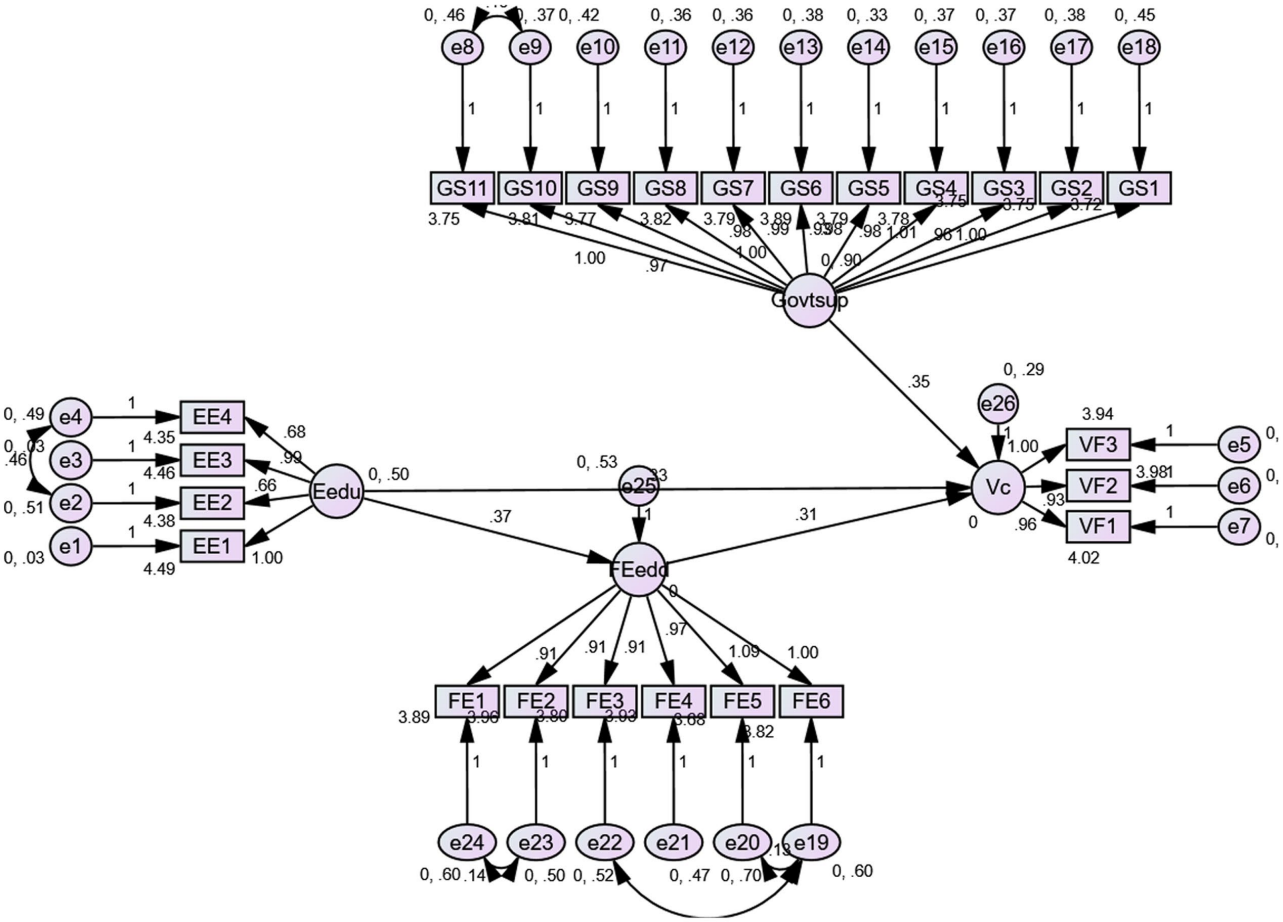
Structural model.

Before testing the hypotheses, we used AMOS version 24.0 to check the structural model’s adaptability. The findings are shown in Figure 3. According to the findings of the hypothesis testing displayed in Table 7, all the variables found the required statistical support. To test our hypotheses, we anticipated that entrepreneurial education has a significant impact on venture capital intention (H1) and discovered that entrepreneurial education has a positive and significant impact on venture capital intention (β = 0.3355, *p* = 0.000); hence, H1 is supported. We also anticipated that entrepreneurial education would significantly impact financial self-efficacy (H2a), and the results show that it did (β = 0.3480, *p* = 0.000); hence, H2a is supported. Furthermore, we anticipated that financial self-efficacy has a significant impact on capital venture intention (H2b), and the study found that financial self-efficacy has a significant impact on capital venture intention. (β = 0.3823, *p* = 0.000), indicating that H2b is also supported.

**Table 7:** Moderated and mediation analyses.

	Relationship	Beta	S.E.	C.R.	*p-*value	Decision
**Direct relationship**						
H1	EE ➔ IVC	0.3355	0.601	5.5852	0.000	Supported
H2a	EE ➔ FSE	0.3480	0.0557	6.2523	0.000	Supported
H2b	FSE ➔ IVC	0.3823	0.0669	5.7174	0.000	Supported
**Moderation effect**						
	**Relationship**	**Beta**	**S.E.**	**C.R.**	***p*-value**	
H3	GS*FSE ➔ IVC	−0.0834	0.0357	−2.3346	0.0206	Supported
**Mediation effect**						
	**Relationship**	**Beta**	**S.E.**	**LLCI**	**ULCI**	
H2	EE ➔ FSE ➔ IVC	0.2927	0.0732	0.1064	0.3996	Supported

### Mediation analysis

For mediation analysis, we anticipated that financial self-efficacy significantly mediates the relationship between entrepreneurial education and venture capital intention. The mediation results shown in Table 7 confirmed that the relationship between entrepreneurial education and intention toward venture capital is significantly mediated by financial self-efficacy (β = 0.2927, LLCI = 0.1064, ULCI = 0.3996). Thus, the H2 is supported.

### Moderation analysis

**Figure 4:**
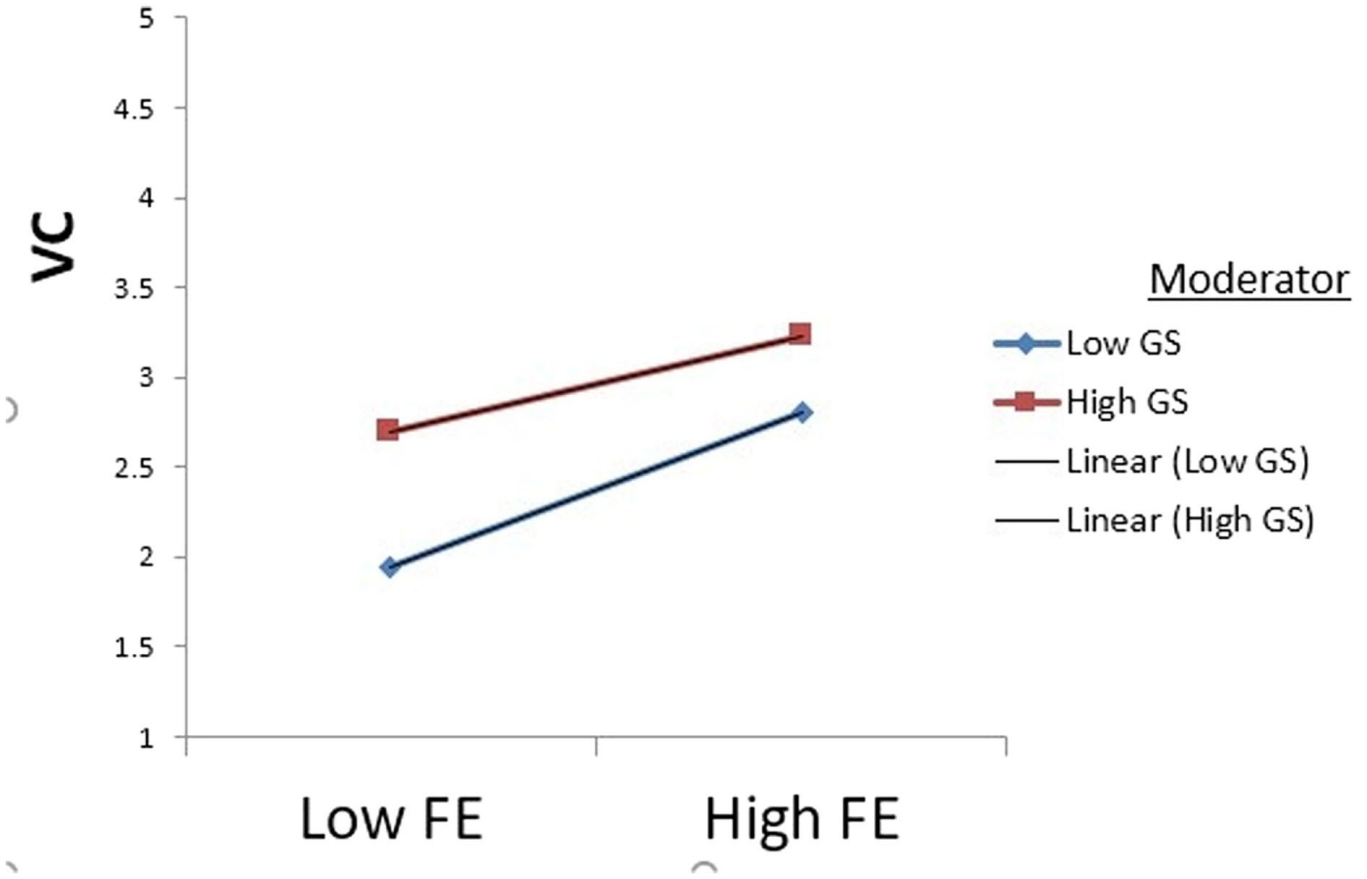
Moderation graph.

We also anticipated that government support significantly moderates the relationship between financial self-efficacy and intention toward venture capital. According to the findings in Figure 4 and Table 7, government support has a moderating effect on the relationship between financial self-efficacy and intention toward venture capital (β = −0.0834, *p* = 0.0206). As a result, H3 is also approved.

## Discussion

This study examines how entrepreneurial education impacts the intention toward venture capital while considering the mediation role of financial self-efficacy and the moderation effect of government support. The findings of the study verify that there exists a significant positive impact of entrepreneurial education on the intention toward venture capital. Moreover, these findings confirm that the students should be equipped with such entrepreneurial education that not only inculcate the ability to come up with some innovative ideas, but also provide information about those platforms that can provide them with financial and intellectual support. This confirms the literature cited above that describes the need for entrepreneurial education that provides students with the knowledge to explore funding sources such as venture capital (Fatoki, 2014; Block et al., 2018). It also verifies that entrepreneurial education relating to financial knowledge enhances the abilities of the students to handle financial challenges and hence improves their financial self-efficacy (Mathisen and Bronnick, 2009; Du et al., 2020; Alferaih, 2022). It is further verified that financial self-efficacy positively impacts intention toward venture capital (McGee et al., 2009). The mediation effect of financial self-efficacy between entrepreneurial education and intention toward venture capital (Zhao et al., 2005; Chen and He, 2011; Kassean et al., 2015) is also verified through the analysis conducted. The role of government in supporting early-stage entrepreneurial ideas to boost them has also been tested by the study and found a significant impact that strengthens the students’ intention to finance their venture through government or private venture capitalists. The initiatives taken by different governments of the world to finance early-stage businesses or the incentives provided by the governments to private venture capitalists for financing the early-stage ventures have given boosts to many of the businesses that by the passage of time have become big business tycoons.

### Theoretical implications

Because the current study established a comprehensive framework, it has major implications for the literature which explains how students’ intention toward venture capital strengthens with government support. Theoretically, this is among the few studies that describe the venture capital intention of university students that is strengthened with government support programs. This study not only examined entrepreneurial education from the business students’ aspect but also enhanced the understanding of how it contributes to fostering the students’ financial self-efficacy rather than directly resulting in venture capital intention. There are numerous pieces of research in the literature that address students’ intention toward venture capital, however, this is the first study that considers government support as a moderator in arousing such intention.

### Practical implications

This study, in particular, provides useful insight into business students’ venture capital ambitions and reaffirms the importance of entrepreneurial education in business courses in developing such intentions. The study will aid the curriculum committee in recognizing the need for education that teaches about entrepreneurship and alternate funding sources. The study will also aid government officials to understand how government support in this aspect can raise the business students’ intention toward venture capital which will ultimately create new business opportunities in the country and new job openings that will help mitigate the employment gap.

### Limitations and future directions

The current study adds significantly to the body of knowledge and practice, but it also has several limitations that could be used to guide future research. First, this study is confined to business students of public and private universities offering business education. Second, this study is limited to the city of Lahore, Pakistan. By expanding the current framework, future research should be conducted in other educational fields as well as in other cities or provinces across the country. Third, this study merely laid the foundation for future venture capital research; however, other financing modes are available to help new startups and are needed to be investigated using the same model. Fourth, the multi-group approach should be used for in-depth analysis. Fifth, the present study developed and tested a moderated mediation model based on financial self-efficacy and government support; in the future, other variables, such as social capital, role models, personality traits, entrepreneurial motivation and entrepreneurial spirit, etc., can be included using mediation and moderation mechanisms for measuring venture capital intention. Finally, rather than focusing on the actual behavior of venture capital financing, the current study concentrated on the development of intention (Neneh, 2022). Future studies can take into account how EE, FSE, and government support influence actual behavior toward venture financing to undertake a more in-depth study since intentions alone might not always dictate actual behavior.

## Conclusion

The current study adds value to the existing literature to get a greater level of intention toward venture capital in the Pakistani setting. The current study examined a moderated mediation model to test the relationship between entrepreneurial education and intention toward venture capital, and the role of financial self-efficacy as a mediator, Moreover, it also examined the moderating role of government support to strengthen the intention toward venture capital. The results indicated that entrepreneurial education positively impacted the intention toward venture capital whereas, Financial self-efficacy has mediated their relationship. Conclusively, students’ financial self-efficacy can be boosted by providing them with education about how to manage financial issues that will increase their intention to acquire finance for their ventures *via* such sources as venture capital. Furthermore, Government support moderated the relationship between financial self-efficacy and intention toward venture capital. This emphasizes the importance of the government’s attention to not only supporting new initiatives but also providing facilities to venture capitalists who assist in the growth of these businesses. As a result, the study has produced evidence from a process viewpoint of how entrepreneurial education leads to a greater desire to venture capital. Furthermore, the study added to the body of the existing knowledge by incorporating financial self-efficacy, which may lead to a desire to invest in venture capital rather than a reinforcement of specific actions. It is also recommended that universities should develop an entrepreneurial eco-system for developing entrepreneurial activities (Feranita et al., 2022).

## Data availability statement

The raw data supporting the conclusions of this article will be made available by the authors, without undue reservation.

## Author contributions

SK: conceptualization, writing—original draft, preparation, methodology, formal analysis, and visualization. HA: preparation, methodology, formal analysis, and visualization. SA: methodology, formal analysis, visualization, and supervision. All authors contributed to the article and approved the submitted version.

## Conflict of interest

The authors declare that the research was conducted in the absence of any commercial or financial relationships that could be construed as a potential conflict of interest.

## Publisher’s note

All claims expressed in this article are solely those of the authors and do not necessarily represent those of their affiliated organizations, or those of the publisher, the editors and the reviewers. Any product that may be evaluated in this article, or claim that may be made by its manufacturer, is not guaranteed or endorsed by the publisher.

## Footnotes

^1^ https://www.worldometers.info/world-population/
