# Supplementary material for: The impact of entrepreneurial education on the propensity of business students to support new ventures: A moderated mediation model
Source: Front Psychol. 2022 Nov 22;13:1046293. doi: 10.3389/fpsyg.2022.1046293 (PMC9723218; doi:10.3389/fpsyg.2022.1046293)
Supplement: Supplementary file 1 [file Data_Sheet_1.docx]

Appendix A

We are conducting a survey on ***the impact of entrepreneurial education on the propensity of business students to support new ventures: A moderated mediation model.*** You are kindly requested to respond to the following statements. Your responses are of great importance as this survey forms a part of our research study. We, therefore, value your cooperation very highly. The survey comprises a different kind of questions. There is no right and wrong answer to the question. We are only interested in your personal opinions. Your responses will be treated in strict confidence and will only be used for research purposes. Thanking you in advance.

**Strongly Agree = 5; Agree = 4; Neutral = 3; Disagree = 2; Strongly Disagree = 1**

| **Items** | **SD** | **D** | **N** | **A** | **SA** |
| --- | --- | --- | --- | --- | --- |
| **Entrepreneurial Education** (Nguyen et al., 2019) | | | | | |
| I am willing to do anything to become an entrepreneur. | **1** | **2** | **3** | **4** | **5** |
| I intend to set up a company in the future. | **1** | **2** | **3** | **4** | **5** |
| I'm not really confident about starting my own business. | **1** | **2** | **3** | **4** | **5** |
| I decided to set up a company in the future. | **1** | **2** | **3** | **4** | **5** |
| **Venture Capital intention** (Baber, 2020) | | | | | |
| I have intention to raise capital for my start-up through venture financing in coming future. | **1** | **2** | **3** | **4** | **5** |
| I predict I would raise capital for my start-up through venture financing in near future. | **1** | **2** | **3** | **4** | **5** |
| I have planned to use venture financing in near future. | **1** | **2** | **3** | **4** | **5** |
| **Government Support** (Korosec and Berman 2006) | | | | | |
| The government provides financial assistance to initiative (Such as subsidies) | **1** | **2** | **3** | **4** | **5** |
| The government supports the initiative in obtaining extra resources (e.g. acquisition, fundraising) | **1** | **2** | **3** | **4** | **5** |
| The government helps the initiative by providing availability to real estate. (buildings) | **1** | **2** | **3** | **4** | **5** |
| The government gives the initiative the opportunity to execute assignments (e.g. waste collection, maintenance of public green spaces, etc.) | **1** | **2** | **3** | **4** | **5** |
| The government supports initiative through the provision of information. | **1** | **2** | **3** | **4** | **5** |
| The government contributes towards initiative awareness | **1** | **2** | **3** | **4** | **5** |
| The government assists in the coordination with other involved parties | **1** | **2** | **3** | **4** | **5** |
| The government encourages collaboration with those parties with an interest in the initiative | **1** | **2** | **3** | **4** | **5** |
| The government provides the initiative with advice, if required. | **1** | **2** | **3** | **4** | **5** |
| The government actively cooperates with the initiative | **1** | **2** | **3** | **4** | **5** |
| The government participates in the initiative by taking responsibility for certain tasks | **1** | **2** | **3** | **4** | **5** |
| **Not at all true= 1 Hardly true= 2 Neutral= 3 Moderately true= 4 Exactly true= 5** | | | | | |
| **Items** | **NT** | **HT** | **N** | **MT** | **ET** |
| **Financial Self Efficacy** (Lown, J.M. 2011) | | | | | |
| It is hard to stick to my spending plan when unexpected expenses arise. | **1** | **2** | **3** | **4** | **5** |
| It is challenging to make progress toward my financial goals. | **1** | **2** | **3** | **4** | **5** |
| When unexpected expenses occur I usually have to use credit. | **1** | **2** | **3** | **4** | **5** |
| When faced with a financial challenge, I have a hard time figuring out a solution. | **1** | **2** | **3** | **4** | **5** |
| I lack confidence in my ability to manage my finances. | **1** | **2** | **3** | **4** | **5** |
| I worry about running out of money in retirement. | **1** | **2** | **3** | **4** | **5** |
